# Exophthalmic Goitre and Its Relation to Diseases of the Thyroid Gland

**Published:** 1894-05-26

**Authors:** A. Maude


### EXOPHTHALMIC GOITRE, AND ITS RELATION TO DISEASE OF THE THYROID GLAND.

By A. Maude.

Exactly fifty years* after Graves' paper on this disease our views are being largely readjusted, and it may be well to take a survey of our present position of knowledge from different points.

The features of this symptom-complex most widely known are the disturbance in the innervation of the heart, with resulting rapid pulse, the exophthalmos, and the enlarged thyroid. No attempt will be made here to dethrone the cardiac symptoms from their position as the most important factors in the disorder. But the purpose of this paper is to bring into relief, for those who have not paid much special attention to this intricate subject, some of the large mass of other important symptoms, and to show how the study of those symptoms confirms the speculations and discoveries recently made in their pathology.

The condition occurs almost entirely among women, either at the commencement of their sexual activity or towards its decline. The earliest symptom noticed by the patient is breathlessness and palpitation, when examination shows a rapid, often irregular, pulse, and a varying, perhaps quite transitory, enlargement of the thyroid.

It is commonly accepted that the thyroid is not necessarily enlarged at all, and that the enlargement is secondary to the cardiac disturbance. I have insisted, however, in a paper read at the London Medical Society in October, that if early cases are watched carefully and frequently some enlargement of the gland will always be found. Other commonly accepted points I also denied in toto, and my views have been confirmed by Professor Greenfield in his Bradshaw Lecture, viz., that there is no true pulsation of the thyroid (any apparent pulsation being only conducted from the great vessels beneath), and that there is no necessary increase in the vascularity of the gland.

It is often a difficult task to determine whether the subject of a goitre is affected with true Graves' disease, because ordinary parenchymatous goitre is frequently associated with anæmia, which may produce some dyspepsia and cardiac irregularity. It therefore becomes of importance to be fully aware of other signs which in my experience are of common occurrence. No attempt will be made to range them in order, and it may be at once pointed out how wide is the area of the nervous system which they affect.

1. "Gastric crises," very similar to those in locomotor ataxy, are frequently found in sudden attacks of sickness and diarrhœa. The latter is very marked in character; it begins suddenly, usually at night, with no apparent dietetic or other cause, lasts two or three days, and then stops equally suddenly. The stools are loose, serous in form, and painless, and may amount to ten or twelve in the day. Both the evacuations and the vomits may contain blood, sometimes even in large quantity.

2. The patient frequently, nay, generally, shows some mental change; they become irritable, morbid, oversensitive to duty, and are often most difficult to manage. These psychoses may become so marked as to amount to distinct melancholia, or mania of an acute type.

3. A valuable and constant symptom is a fine, regular tremor. It may affect almost all the skeletal muscles, though very rarely the face. It is most marked, however, in the flexor and extensor muscles of the arms, producing a small oscillation of the whole hand in one plane. The interossei are not affected as they are in alcoholism, and the tongue always escapes.

4. An extreme muscular weakness is the rule rather than the exception. Not only are these people readily fatigued, but the paresis of the legs is so great as to render them utterly bedridden, sometimes for long periods. They have also the same tendency to give way at the knee in walking that we see in locomotor ataxy. The form of spinal inco-ordination recently described as astasia-abasia is also quite common. Various forms of paralysis occur, though they are rare. Weakness of the facial muscles, however, is the rule. Forms of ophthalmoplegia of the third, fourth, and sixth nerves have often been described. The "stare," so marked a feature in the disease, is due to two causes, first, but least often, to true exophthalmos. This is rarely seen, in my experience, except in old-standing cases. The protrusion of the globe is due either to a venous congestion of the orbit, or to an accumulation of orbital fat.

Secondly, to retraction of the lids. The writer endeavoured to prove some years ago that this retraction is the result of a semi-paralytic state of the circular muscle of the eyelids (orbicularis palpebrarum). This retraction of the lids is known as "Stellwag's sign," from the distinguished German who first described it. There is frequently also a peculiar slow movement of the upper lids in the act of closing them, so that if the eyes are made to turn slowly downwards, the lids, instead of following at the same rate as the eyeballs move, descend much too slowly, and seem to stick halfway. This is called Von Graefe's sign, because the great oculist first described it. Stellwag's sign is, however, found in many persons in poor health, particularly neurotic persons, who have not got Graves' disease.

5. The skin often presents changes in pigmentation, generally becoming darker in certain positions, notably round the eyes, in the armpits, and wherever the clothing is tight, as under the stays or garters. Occasionally, however, patches devoid of pigment occur. The hair always becomes thin and weak, and entire baldness may result. The hair may be thus affected all over the body. Flushing and copious sweats are common. One poor patient of the writer was found in the severe winter of 1891-2 frozen stiff in her sheets after the excessive sweating of the night, which in most cases comes on in the early morning.

6. The uterine functions are frequently much disturbed, the patients having either amenorrhœa or over profuse menstruation. I have, however, not found these conditions so common as older writers on the subject would infer. Pregnancy often seems to exert a good influence, and the late M. Charcot used to say would often cure it.

7. Evidence has been gradually accumulating since 1885 to show that there is a connection between

* It had been described in 1786 by Hillier Parry, of Bath, but his account, though better than Graves', was not published till after his death, and attracted no attention.

myxœdema and Graves' disease. The two states may co-exist in the same patient, but the common history is that in the early stages of myxœdema an enlarged thyroid with symptoms of Graves' disease are present. I have one patient, having Graves' disease, whose mother is myxœdematous. The daughter's goitre is large, and contains apparently a large amount of firm, almost hard, fibrous tissue. It will be interesting to note if this fibrous tissue increases at the expense of the true gland tissue. If so, myxœdema may ensue, for Professor Greenfield's specimens show that in myxœdema there is a marked degeneration of the true thyroid elements into fibrous issue.

*(To be continued.)*

